# Lipopolysaccharide Derived From the Lymphoid-Resident Commensal Bacteria *Alcaligenes faecalis* Functions as an Effective Nasal Adjuvant to Augment IgA Antibody and Th17 Cell Responses

**DOI:** 10.3389/fimmu.2021.699349

**Published:** 2021-07-01

**Authors:** Yunru Wang, Koji Hosomi, Atsushi Shimoyama, Ken Yoshii, Takahiro Nagatake, Yukari Fujimoto, Hiroshi Kiyono, Koichi Fukase, Jun Kunisawa

**Affiliations:** ^1^ Laboratory of Vaccine Materials, Center for Vaccine and Adjuvant Research, and Laboratory of Gut Environmental System, National Institutes of Biomedical Innovation, Health and Nutrition (NIBIOHN), Ibaraki, Japan; ^2^ Graduate School of Pharmaceutical Sciences, Osaka University, Suita, Japan; ^3^ Department of Chemistry, Graduate School of Science, Osaka University, Toyonaka, Japan; ^4^ Project Research Center for Fundamental Sciences, Osaka University, Toyonaka, Japan; ^5^ Institute for Radiation Sciences, Osaka University, Suita, Japan; ^6^ Graduate School of Medicine, Osaka University, Suita, Japan; ^7^ Department of Chemistry, Faculty of Science and Technology, Keio University, Yokohama, Japan; ^8^ International Research and Development Center for Mucosal Vaccines, The Institute of Medical Science, The University of Tokyo, Tokyo, Japan; ^9^ IMSUT Distinguished Professor Unit, The Institute of Medical Science, The University of Tokyo, Tokyo, Japan; ^10^ Graduate School of Medicine, Chiba University, Chiba, Japan; ^11^ Department of Medicine, School of Medicine and CU-UCSD Center for Mucosal Immunology, Allergy and Vaccine, University of California San Diego, La Jolla, CA, United States; ^12^ Graduate School of Dentistry, Osaka University, Suita, Japan; ^13^ Department of Microbiology and Immunology, Graduate School of Medicine, Kobe University, Hyogo, Japan; ^14^ Research Organization for Nano & Life Innovation, Waseda University, Tokyo, Japan

**Keywords:** *Alcaligenes faecalis*, nasal vaccine, lipopolysaccharide, T helper 17 cell, adjuvant

## Abstract

*Alcaligenes* spp., including *A. faecalis*, is a gram-negative facultative bacterium uniquely residing inside the Peyer’s patches. We previously showed that *A. faecalis*-derived lipopolysaccharides (*Alcaligenes* LPS) acts as a weak agonist of toll-like receptor 4 to activate dendritic cells and shows adjuvant activity by enhancing IgG and Th17 responses to systemic vaccination. Here, we examined the efficacy of *Alcaligenes* LPS as a nasal vaccine adjuvant. Nasal immunization with ovalbumin (OVA) plus *Alcaligenes* LPS induced follicular T helper cells and germinal center formation in the nasopharynx-associated lymphoid tissue (NALT) and cervical lymph nodes (CLNs), and consequently enhanced OVA-specific IgA and IgG responses in the respiratory tract and serum. In addition, nasal immunization with OVA plus *Alcaligenes* LPS induced OVA-specific T cells producing IL-17 and/or IL-10, whereas nasal immunization with OVA plus cholera toxin (CT) induced OVA-specific T cells producing IFN-γ and IL-17, which are recognized as pathogenic type of Th17 cells. In addition, CT, but not *Alcaligenes* LPS, promoted the production of TNF-α and IL-5 by T cells. Nasal immunization with OVA plus CT, but not *Alcaligenes* LPS, led to increased numbers of neutrophils and eosinophils in the nasal cavity. Together, these findings indicate that the benign nature of *Alcaligenes* LPS is an effective nasal vaccine adjuvant that induces antigen-specific mucosal and systemic immune responses without activation of inflammatory cascade after nasal administration.

## Introduction

Commensal bacteria in the gut are involved in the regulation of host immunity; therefore, they are expected to play important roles not only in host immune responses to immunization but also in host responses to pathogenic infection. Indeed, accumulating evidence has already indicated the involvement of certain commensal bacteria in the regulation of specific immunity. For instance, *Klebsiella* spp. have been shown to induce T helper 1 (Th1) cell polarization, and segmented filamentous bacteria (SFB) have been shown to drive Th17 cell responses to pathogenic infection (1, 2). Similarly, *Clostridium* spp. have been shown to induce regulatory T cells for the control of allergic diseases (3).

Previously, we demonstrated that commensal bacteria are present not only in the intestinal lumen but also inside intestinal tissues, such as Peyer’s patches (PPs) and the colonic lamina propria (4). For the first time, we found that the gram-negative bacterium *Alcaligenes* spp. including *A. faecalis*, is a representative bacterium that symbiotically resides in PPs. Our previous study showed that *A. faecalis* promotes the production of several cytokines (e.g., transforming growth factor beta [TGF-β], B-cell activating factor [BAFF], and interleukin 6 [IL-6]) by dendritic cells (DCs) to enhance the production of IgA in the intestine (4). A subsequent study revealed that *A. faecalis* increases IL-10 producing DCs, which contributes to establish the symbiotic environment in the gut (5). A more recent study by our group using *A. faecalis* revealed that lipopolysaccharides (LPS) derived from *A. faecalis* (*Alcaligenes* LPS) possesses unique immunomodulatory activity. Indeed, *Alcaligenes* LPS enhanced the production of IL-6 from DCs by acting as a weak agonist of toll-like receptor 4 (TLR4) (6). Of note, the biological activity of *Alcaligenes* LPS, when administered to OVA by subcutaneous injection in mice, was lower than that of *E. coli*-derived LPS (*E. coli* LPS). In addition, *Alcaligenes* LPS was able to enhance both antigen-specific IgG production and Th17 responses without inducing excessive inflammation. These findings suggest the potential of *Alcaligenes* LPS as a novel vaccine adjuvant (6).

Although subcutaneous or intramuscular injection of vaccines is commonly accepted and practiced, mucosal vaccination (e.g., nasal and oral vaccines) has currently attracted attention because of several advantages, including reduced fear and pain, decreased medical waste, such as syringe and needle, and abatement of the work of medical staff responsible for vaccination. In addition, mucosal vaccination has the benefit of inducing both systemic and mucosal immune responses (7, 8). After nasal immunization, nasopharynx-associated lymphoid tissue (NALT) is one of the responsible sites for inducing antigen-specific immune responses. NALT is located at the bottom edge of nasal cavity in rodents (9), and the human tonsils known as Waldeyer’s tonsillar ring are considered as equivalent lymphoid tissues to rodent NALT (10). NALT has all the necessary immunocompetent cells, such as B cells, T cells, DCs, and M cells, to initiate antigen-specific immune responses (11). M cells located in the NALT epithelium act as antigen uptake cells to deliver antigens to DCs (11). The DCs then process and present these antigens to T cells and B cells in germinal centers (GC) located in the NALT to initiate antigen-specific IgA responses (11, 12). IgA class switching recombination (CSR) is a critical step for promoting IgA^+^ B cell development in the GC of NALT with the essential support by follicular T helper cells (Tfh cells) (12, 13). The antigen-specific IgA produced by IgA^+^ B cells is secreted through the epithelium into the nasal cavity, where it binds to antigens to prevent the invasions of pathogens from nasal cavity (11).

Despite these advantages of mucosal vaccination, one of the issues to be resolved includes the induction of immune tolerance to cause immune nonresponsiveness (14). In this regard, mucosal adjuvants are required for induction of mucosal antigen-specific immune responses without inducing immune tolerance. Recently, some adjuvant candidates for nasal vaccines have been developed by using microbial components (12). For example, when the TLR5 agonist, flagellin of *Salmonella typhimurium*, is used as a nasal adjuvant for the H7N9 influenza subunit vaccine, it can induce effective IgG and IgA antibody responses, Th1 and Th2 responses (15). Also, intranasal co-administration of adenylate cyclase toxin of *Bordetella pertussis* and pertactin elicits robust IgG and IgA antibody responses and has a protective effect when challenged with *B. pertussis* intranasally (16).

Here, we evaluated the efficacy of *Alcaligenes* LPS as an adjuvant when administered to mice by nasal immunization. We found that *Alcaligenes* LPS induced both systemic and mucosal immune responses, including antigen-specific IgG and IgA antibody production as well as Th17 responses, without inducing inflammation locally, confirming the potential of *Alcaligenes* LPS as a nasal adjuvant.

## Materials and Methods

### Mice

Female BALB/c mice (age 8–9 weeks) were purchased from CLEA Japan, Inc. (Tokyo, Japan). The mice were kept in a specific-pathogen-free (SPF) environment on a 12/12-h light/dark cycle at the National Institutes of Biomedical Innovation, Health, and Nutrition (Osaka, Japan). All experimental procedures were performed in accordance with the guidelines of the Animal Care and Use Committee of the National Institutes of Biomedical Innovation, Health, and Nutrition (Approval Nos. DS27-47R13 and DS27-48R13).

### Preparation of LPS

*Alcaligenes* LPS was prepared as described previously (6). Briefly, *Alcaligenes* LPS was extracted from heat-killed (60°C for 30 min) *A. faecalis* (13111T, Biological Resource Center, NITE [NBRC], Japan) by using an LPS Extraction Kit (iNtRON Biotechnology, Inc., Sangdaewon-Dong, Korea). After extraction, *Alcaligenes* LPS is lyophilized and stored as a powder at -30°C, and the weight was measured by using Semi-Micro Analytical Balances (GR-202; AND company, Tokyo, Japan). For stock solution, the LPS was added to phosphate-buffered saline (PBS; Nacalai Tesque, Inc., Kyoto, Japan) to a concentration of 1 mg/ml, sonicated for 5 min, and then stored at −30°C until use.

### Immunization

Immunization was performed as described previously (17). Briefly, on days 1, 7, and 17, the mice were intranasally immunized with 5 μg of ovalbumin (OVA) (Sigma-Aldrich) alone or 10 μg of *Alcaligenes* LPS or 1 μg of cholera toxin (CT) isolated from *Vibrio cholerae* (List Biological Laboratories, Campbell, CA, USA) in 15 µL of PBS and administered as 7.5 µL in each nostril of mice without anesthesia. One week after the final immunization, nasal wash, bronchoalveolar lavage fluid (BALF), serum, nasal passage, NALT, cervical lymphoid nodes (CLNs), and spleen were collected as previously described (17, 18) and used for analysis.

### Enzyme-Linked Immunosorbent Assay (ELISA)

ELISA was performed as described previously (19). The bottom of flat-bottom 96-well immunoplates (Thermo Fisher Scientific Inc., Waltham, MA, USA) were coated with OVA diluted in PBS to a concentration of 1 mg/ml and then the plates were incubated overnight at 4°C. After incubation, the plates were blocked with 1% (w/v) bovine serum albumin (BSA; Nacalai Tesque, Inc.) in PBS for 2 h at room temperature. After blocking, the plates were washed three times with PBS containing 0.05% (v/v) Tween 20 (Nacalai Tesque, Inc.).

Next, serum, nasal wash, or bronchoaveolar lavage fluid (BALF) samples were serially diluted with 1% (w/v) BSA, containing 0.05% (v/v) Tween 20 in PBS and seeded into the plates; the plates were then incubated for 2 h at room temperature and washed three times with PBS containing 0.05% Tween 20. After washing, horseradish peroxidase-conjugated goat anti-mouse IgG or IgA (Southern Biotech, Inc., Birmingham, AL, USA) diluted with 1% (w/v) BSA containing 0.05% (v/v) Tween 20 in PBS was added to the plates and left to react for 1 h at room temperature. The plates were subsequently washed three times with PBS containing 0.05% Tween 20. Tetramethylbenzidine peroxidase substrate (SeraCare Life Sciences Inc., Milford, MA, USA) was then added, and the plates were left to react for 2 min at room temperature; 0.5 N HCl (Nacalai Tesque, Inc.) was added to stop the reaction. Absorbance at 450 nm was measured by using an iMark™ Microplate Absorbance Reader (Bio-Rad Laboratories, Inc., Hercules, CA, USA).

### Immunohistochemistry

Immunohistological analysis was performed as described previously (17). NALT and CLNs were embedded in Tissue-Tek O.C.T. Compound (Sakura Finetek Japan Co., Ltd., Tokyo, Japan) to make frozen blocks. Blocks were frozen by liquid nitrogen and stored at −80°C until use. Sections (6-μm-thick) of NALT and CLNs were cut at 20°C by using a Leica CM3050 S cryostat (Leica Biosystems, Nussloch, Germany). Then, the sections of NALT and CLNs were air-dried, fixed with 100% acetone (Nacalai Tesque, Inc.) for 1 min, and washed 2 times with PBS for 5 min each time. After washing, the sections were blocked with 2% Newborn Calf Serum (NCS; Equitech-Bio, Kerrville, TX, USA) -PBS for 30 min, stained with purified anti-B220 antibody (BioLegend, San Diego, CA, USA) and biotin-PNA (Vector Laboratories, Inc., Burlingame, CA, USA) and incubated overnight at 4°C. After incubation, the sections were washed two times with PBS for 5 min each time, stained with Cy3-labeled anti-hamster IgG (Jackson ImmunoResearch Inc., West Grove, PA, USA) and Alexa Fluor 488/Streptavidin Conjugate (Invitrogen, Thermo Fisher Scientific Inc.) for 30 min, washed two times with PBS for 5 min each time, stained with DAPI (AAT Bioquest, Inc., Sunnyvale, CA, USA) for 10 min, and washed two times with PBS for 5 min each time. Finally, each section was covered with one drop of Fluoromount (Diagnostic BioSystems, Pleasanton, CA, USA) followed by a 24 × 36-mm-thick cover glass (Matsunami Glass USA Inc., Bellingham, WA, USA) and observed under a BZ-9000 BioRevo fluorescence microscope (Keyence Corp., Osaka, Japan).

### T-Cell Assay

T-cell assay was performed as described previously (17). Cell suspension collected from CLNs and spleen was passed through a 100-μm cell filter (Thermo Fisher Scientific Inc.); then mixed with red blood cell lysis buffer (1.5 M NH_4_Cl, 100 mM KHCO_3_, and 10 mM EDTA-2Na [all Nacalai Tesque, Inc.]) for 1 min at room temperature; the resulting suspension was passed through a 100-μm cell filter (Thermo Fisher Scientific Inc.) again, and the filtrate was retained. CD4^+^ T cells were purified from the filtrate by using CD4 (L3T4) MicroBeads and a magnetic cell separation system (Miltenyi Biotec, Bergisch Gladbach, North Rhine-Westphalia, Germany). Splenic cells were treated with 30 Gy of ionizing radiation and used as antigen-presenting cells (APCs). The CD4^+^ T cells (2 × 10^5^ cells/well) and APCs (1 × 10^4^ cells/well) were suspended in RPMI1640 medium (Sigma-Aldrich) supplemented with 10% fetal bovine serum (FBS; Life Technologies, Thermo Fisher Scientific Inc.), 1 mM sodium pyruvate solution (Nacalai Tesque, Inc.), 1% penicillin–streptomycin mixed solution (Nacalai Tesque, Inc.), and 0.5 mM 2-mercaptoethanol (Gibco, Thermo Fisher Scientific Inc.); seeded in round-bottom 96-well plates (Thermo Fisher Scientific Inc.); and cultured with or without 1 mg/ml OVA for 72 h. The number of viable cells was determined by using a CyQUANT Cell Proliferation Assay kit (Invitrogen, Thermo Fisher Scientific Inc.), and the absorbance of the cells was measured at 485/535 nm with an ARVO X2 (PerkinElmer, Yokohama, Japan) fluorescence microplate reader. The culture supernatant was collected and used for the measurement of the concentrations of the cytokines as follows: interferon gamma (IFN-γ), IL-4, IL-17, IL-10, and TNF-α were determined by using a BD CBA Mouse Th1/Th2/Th17 Cytokine Kit (BD Biosciences, San Jose, CA, USA), and the concentration of IL-5 was determined by using IL-5-specific ELISA kit (BioLegend).

### Flow Cytometric Analysis

Flow cytometry was performed as previously described (20). Cells were collected from NALT and CLNs and incubated with 5 μg/ml anti-CD16/32 antibody (TruStain FcX; BioLegend) and 7-AAD viability staining solution (BioLegend) for 15 min at room temperature to avoid non-specific staining and to detect dead cells, respectively. Then, the cells were stained with the following fluorescently labeled antibodies for 30 min at 4°C: GC and IgM^−^ IgA^+^ B cells were stained with FITC-IgA (BD Biosciences; clone: C10-3), PE-Cy7-IgM (BioLegend; clone: RMM-1), AF647-GL7 (BioLegend; clone: GL7), and BV421-B220 (BioLegend; clone: RA3-6B2). Follicular T helper cells were stained with FITC-CD3ϵ (BD Biosciences; clone: 145-2C11), PE-PD-1 (BioLegend; clone: 29F.1A12), APC-Cy7-CD8α (BioLegend; clone: 53-6.7), and BV421-CD4 (BioLegend; clone: RM4-5). Neutrophils and eosinophils were stained with FITC-Ly6G^+^ (BioLegend; clone: 1A8), APC-Cy7-CD11b (BioLegend; clone: M1/70), BV421-Siglec-F (BD Biosciences; clone: E50-2440), and APC-CD45 (BioLegend; clone: 30-F11).

Intracellular cytokine staining was performed as previously described with modification (21, 22). Cells were collected from mice spleen were stimulated with 50 ng/ml phorbol 12-myristate 13-acetate (PMA; Sigma-Aldrich) and 750 ng/ml ionomycin (Sigma-Aldrich) for 4 h at 37°C; 5 ng/ml brefeldin A (BioLegend) was added at around 3^rd^ hour. After incubation, the cells were stained with NIR-zombie (BioLegend), FITC-TCR-β (BioLegend; clone: H57-597), PerCP-CD4 (BioLegend; clone: GK1.5), and BV421-CD45 (BioLegend; clone: 30-F11). The cells were fixed and permeabilized by using BD Cytofix/Cytoperm plus (BD Biosciences) and then stained with PE-IFN-γ (BioLegend; clone: XMG1.2) and AF647-IL-17A (BD Biosciences; clone: TC11-18H10). Samples were examined with a MACSQuant Analyzer (Miltenyi Biotec) and the data were analyzed using FlowJo software v.10.2 (BD Biosciences).

### Measurement of Lymphocytes in Blood

The numbers of lymphocytes in the blood were enumerated as previously described (23). Briefly, blood samples (100 μl) mixed with 1.5 μl of 10 mM EDTA-2Na (Nacalai Tesque, Inc.) were diluted 1:6 with saline solution (Otsuka Pharmaceutical Co., Ltd., Tokyo, Japan) for measuring the number of lymphocytes with a Vet Scan HMII hematology analyzer (Abaxis, Union City, CA, USA).

### Statistical Analysis

Data are presented as mean ± SD. Statistical analyses were performed by using one-way ANOVA with the Bonferroni post-hoc test using PRISM 6 software (GraphPad Software, San Diego, CA, USA).

## Results

### Nasally Co-Administered *Alcaligenes* LPS Promotes Respiratory Antigen-Specific IgA Antibody Production

Previously, we demonstrated *in vitro* that *Alcaligenes* LPS enhanced IgA production by B cells co-cultured with DCs (6). We extended our previous study by investigating the efficacy of *Alcaligenes* LPS as an adjuvant for nasal vaccination *in vivo*. To determine the optimal dose of *Alcaligenes* LPS, mice were nasally immunized with OVA alone (Mock group), OVA plus 1 or 10 μg of *Alcaligenes* LPS or *E. coli* LPS. Nasal immunization with 10 μg of *Alcaligenes* LPS resulted in the induction of higher levels of nasal IgA responses than 1 μg of *Alcaligenes* LPS (**Supplementary Figure 1**); therefore, we determined the 10 μg of *Alcaligenes* LPS for the nasal immunization in this study. We further confirmed that 10 μg of *E. coli* LPS also increased OVA-specific IgA production in the nasal wash (**Supplementary Figure 1**); however, mice receiving LPS from *E. coli*, but not *Alcaligenes*, showed severe side effects, such as lymphopenia (**Supplementary Figure 2**), which is consistent with our previous study (6). Therefore, we decided to employ another control, cholera toxin (CT), a gold standard experimental mucosal adjuvant.

**Figure 1:**
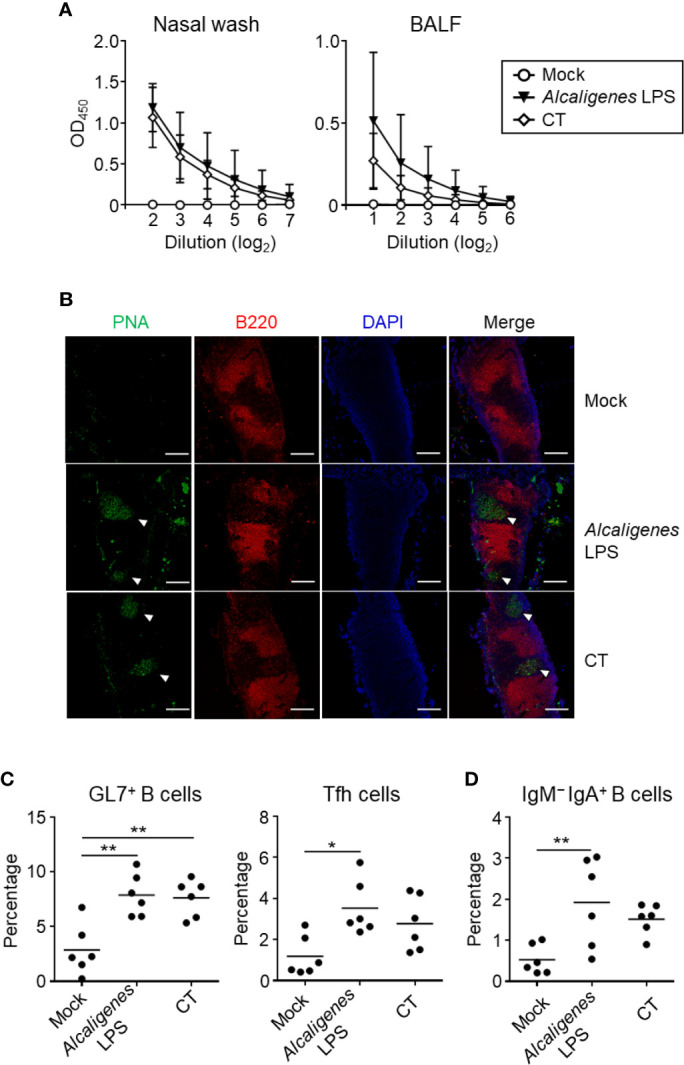
*Alcaligenes* LPS promotes mucosal OVA-specific IgA antibody production upon nasal immunization. Mice were nasally immunized three times with OVA alone (Mock) or with OVA plus *Alcaligenes* LPS or CT; one week after the final immunization, **(A)** Nasal wash and BALF were collected to determine levels of OVA-specific IgA by ELISA. **(B)** Formation of GC in NALT were observed by immunohistochemical analysis. PNA: GC marker; B220: B cell marker; arrow heads: GC location. Induction of GC GL7^+^ B cells (gated on: CD3ϵ^−^ B220^+^ GL7^+^) and Tfh cells (gated on: CD3ϵ^+^ CD8α^−^ CD4^+^ PD-1^+^) **(C)** and of IgM^−^ IgA^+^ B cells (gated on: CD3ϵ^−^ B220^+^ GL7^+^ IgM^−^ IgA^+^) **(D)** in NALT were analyzed by flow cytometry (*n* = 5 or 6 per group). Data are representative of two independent experiments and statistical significance was determined by one-way ANOVA (*p < 0.05; **p < 0.01).

To examine OVA-specific IgA production in the nasal wash and BALF, mice were nasally immunized with OVA alone (Mock group), OVA plus 10 μg of *Alcaligenes* LPS (*Alcaligenes* LPS group) or 1 μg of CT (CT group). We found that the nasal wash and BALF from Mock group contained practically no sign to undetectable levels of OVA-specific IgA (**Figure 1A**). In contrast, the nasal wash and BALF from *Alcaligenes* LPS group contained substantial levels of OVA-specific IgA antibody, which was compatible to the levels seen in the CT group (**Figure 1A**).

The production of IgA antibody is associated with B cell class-switch recombination from IgM to IgA in the GC of NALT, which is supported by Tfh cells (11). Immunohistological analysis revealed the formation of GC in the NALT was observed in both groups of mice nasal immunized with OVA plus *Alcaligenes* LPS or CT, but not Mock group (**Figure 1B**). Consistent with this finding, flow cytometry analysis demonstrated that the increased percentage of GC GL7^+^ B cells were found in both *Alcaligenes* LPS and CT groups when compared to Mock group (**Figure 1C**). In addition, the percentage of PD-1^+^ Tfh cells (**Figure 1C**) and IgM^−^ IgA^+^ B cells (**Figure 1D**) were significantly increased in the NALT from *Alcaligenes* LPS group compared with that in Mock group. Together, these results indicate that *Alcaligenes* LPS promoted the formation of GC in the NALT with Tfh cells and IgA^+^ B cells for the subsequent IgA antibody production in the respiratory tract.

### *Alcaligenes* LPS Promotes Systemic Antibody Responses

**Figure 2:**
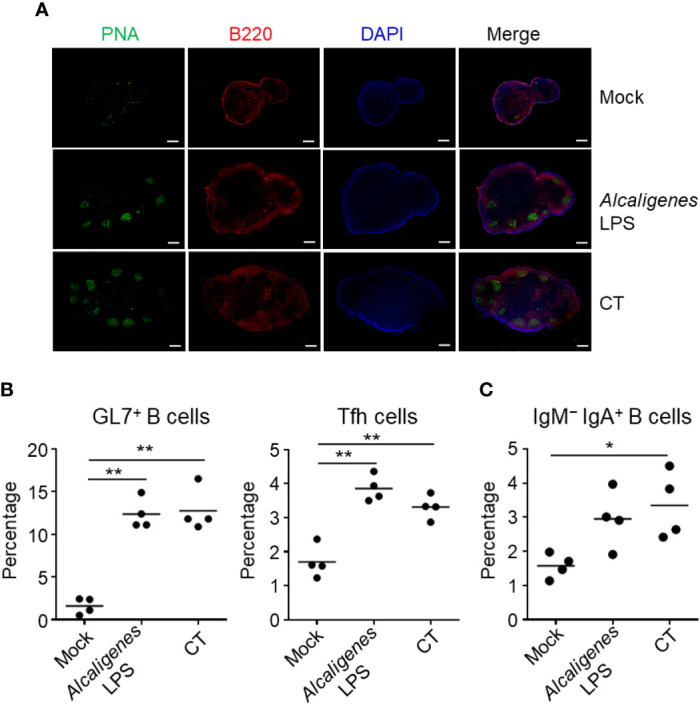
*Alcaligenes* LPS induces germinal center formation in CLNs. Mice were nasally immunized three times with OVA alone (Mock) or with OVA plus *Alcaligenes* LPS or CT; one week after the final immunization, GC formation **(A)**, and the induction of GC GL7^+^ B cells (gated on: CD3ϵ^−^ B220^+^ GL7^+^) and Tfh cells (gated on: CD3ϵ^+^ CD8α^−^ CD4^+^ PD-1^+^) **(B)** and of IgM^−^ IgA^+^ B cells (gated on: CD3ϵ^−^ B220^+^ GL7^+^ IgM^−^ IgA^+^) **(C)** in CLNs were examined by flow cytometry. (*n* = 4 per group). Data are representative of two independent experiments and were analyzed by one-way ANOVA (*p < 0.05; **p < 0.01).

We examined the immune responses in the CLNs, which are lymph nodes that drain the nose. As in the NALT, GC formation together with significantly increased or higher induction of GL7^+^ B cells, Tfh cells, and IgM^−^ IgA^+^ B cells were detected in the CLNs from *Alcaligenes* LPS or CT group when compared to the Mock group (**Figures 2A–C**).

**Figure 3:**
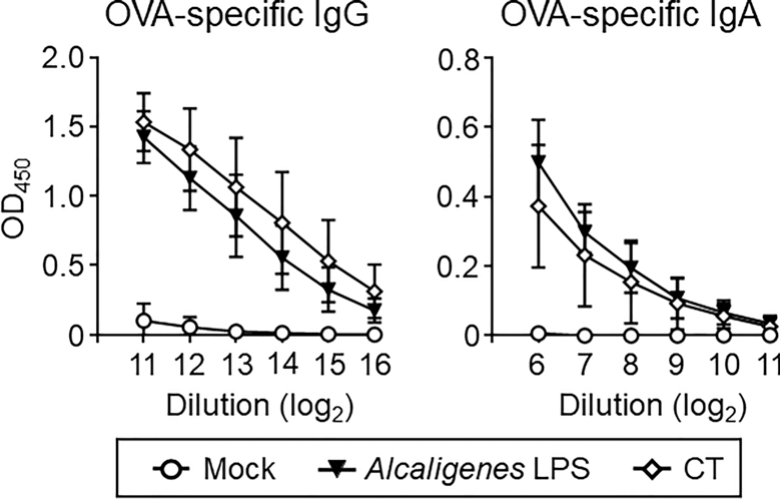
*Alcaligenes* LPS promotes systemic antibody responses. Mice were nasally immunized three times with OVA alone (Mock) or with OVA plus *Alcaligenes* LPS or CT; one week after the final immunization, serum was collected to determine OVA-specific IgG and IgA by ELISA (*n* = 5 per group). Data are representative of two independent experiments.

To further assess whether nasally co-administered *Alcaligenes* LPS also supports the induction of antigen-specific systemic antibody responses or not, serum antibodies were examined. Higher levels of OVA-specific IgG and IgA responses were noted in the serum from *Alcaligenes* LPS group, which were almost comparable with CT group (**Figure 3**). On the other hand, the Mock group’s antibody responses were negligible (**Figure 3**). Thus, co-administered *Alcaligenes* LPS could also support the elevated antigen-specific systemic antibody responses through nasal vaccination.

### *Alcaligenes* LPS Promotes an OVA-Specific Th17 Cell Response

**Figure 4:**
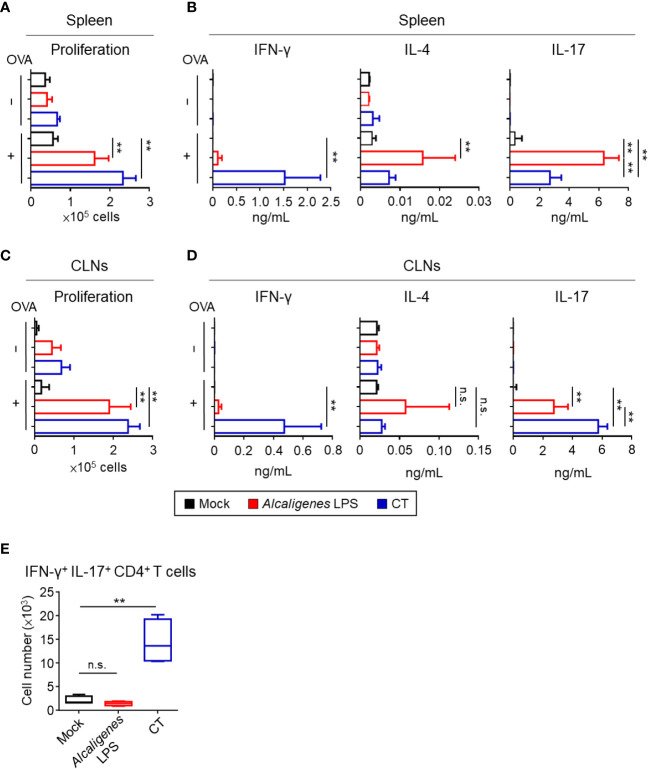
*Alcaligenes* LPS promotes OVA-specific Th17 response. Mice were nasally immunized three times with OVA alone (Mock) or with OVA plus *Alcaligenes* LPS or CT; one week after the final immunization, splenic or CLNs CD4^+^ T cells were collected and stimulated with OVA by *ex vivo*. **(A)** Proliferation of splenic CD4^+^ T cells was determined by CyQUANT^®^ Cell Proliferation Assays Kits and fluorescence microplate reader, ARVO X2 with measuring at 485/535 nm. **(B)** Production of cytokines: IFN-γ, IL-4 and IL-17A in the supernatant of splenic CD4^+^ T cell culture was collected and measured by the CBA kit. **(C)** Proliferation of CLNs CD4^+^ T cells. **(D)** Production of cytokines: IFN-γ, IL-4 and IL-17A in the supernatant of CLNs CD4^+^ T cell culture. **(E)** Number of IFN-γ^+^ IL-17^+^ CD4^+^ T cells (gated on: zombie^−^ CD45^+^ TCR-β^+^ CD4^+^ IFN-γ^+^ IL-17^+^) in spleen were analyzed by intracellular cytokine staining and flow cytometry (*n* = 6 per group). Data are representative of two independent experiments and were analyzed by one-way ANOVA (**p < 0.01; n.s., not significant).

We examined T cell responses, such as cell proliferation and cytokine production in the spleen and CLNs. CD4^+^ T cells from the spleen and CLNs of mice nasally immunized with OVA plus *Alcaligenes* LPS or CT proliferated vigorously upon the *in vitro* stimulation with OVA, when compared with those from the Mock group (**Figures 4A, C** **)**. The finding suggests that *Alcaligenes* LPS is a potent adjuvant for the enhancement of CD4^+^ T cell responses.

Next, we examined the production of cytokines from OVA-specific CD4^+^ T cells, especially related to the Th1 (IFN-γ), Th2 (IL-4), and Th17 (IL-17). Consistent with low OVA-induced CD4^+^ T cell proliferation activity of the Mock group from spleen and CLNs, negligible to low amounts of cytokines were noted (**Figures 4B, D** **)**. In contrast, splenic and CLNs CD4^+^ T cells from the *Alcaligenes* LPS group preferentially produced IL-17 with little production of IL-4 and IFN-γ, whereas the CT group showed significantly increased production of both IFN-γ and IL-17 with less production of IL-4 (**Figures 4B, D** **)**.

It has been considered that T cells secreting IL-17 alone are considered non-pathogenic and contribute to immunological defense against extracellular pathogens, whereas T cells producing both IL-17 and IFN-γ are pathogenic to cause inflammation and autoimmunity (24, 25). One of the differences between *Alcaligenes* LPS and CT groups was the significantly higher IFN-γ production in the CT group. Flow cytometric analysis revealed that splenic CD4^+^ T cells from the CT group contained significantly higher numbers of IFN-γ^+^ IL-17^+^ CD4^+^ T cells compared with that in the Mock or *Alcaligenes* LPS group (**Figure 4E**). These results indicate that nasally co-administered *Alcaligenes* LPS primarily induced Th17 cell-mediated non-pathogenic responses, whereas nasally co-administered CT induced pathogenic Th17 cell responses.

### *Alcaligenes* LPS Has Low Inflammatory but High Regulatory Properties

**Figure 5:**
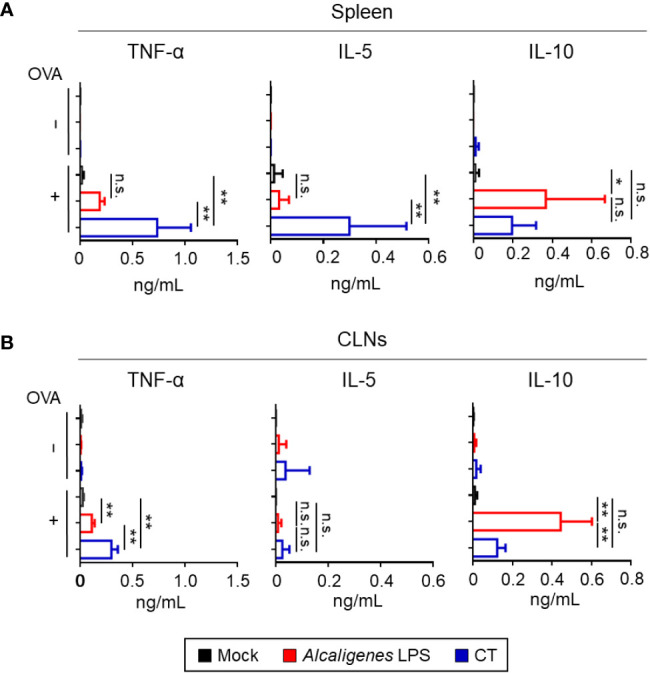
*Alcaligenes* LPS has low Inflammatory but high regulatory properties. Mice were nasally immunized three times with OVA alone (Mock) or with OVA plus *Alcaligenes* LPS or CT. One week after the final immunization, the production of TNF-α, IL-5, and IL-10 in the spleen **(A)** and CLNs **(B)** of nasally immunized mice was surveyed after *ex vivo* stimulation by OVA *(n* = 6 per group). Data are representative of two independent experiments and were analyzed by one-way ANOVA (*p < 0.05; **p < 0.01; n.s., not significant).

In addition to classical Th subsets associated with cytokines examined above, T cells are also known to produce various inflammatory and regulatory cytokines (26). Therefore, we examined other cytokine production profiles (e.g., TNF-α, IL-5, and IL-10) by OVA-specific CD4^+^ T cells from CLNs and spleen of *Alcaligenes* LPS group. Significantly increased TNF-α and IL-5 production were noted in the cultures containing splenic and CLNs CD4^+^ T cells from the CT group, but not in the Mock and *Alcaligenes* LPS groups. It is interesting to note that the production of IL-10 was preferentially heightened in splenic and CLNs CD4^+^ T cell cultures from the *Alcaligenes* LPS group (**Figures 5A, B** **)**.

**Figure 6:**
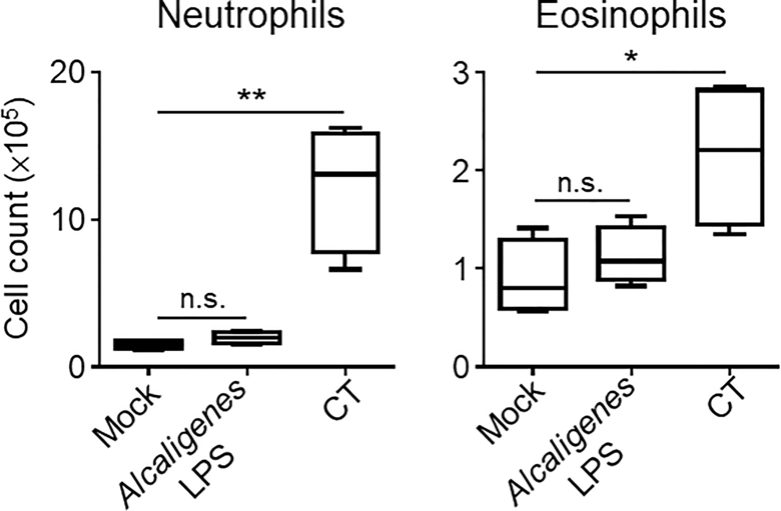
*Alcaligenes* LPS induced little inflammation at the site of administration. Mice were nasally immunized three times with OVA alone (Mock) or with OVA plus *Alcaligenes* LPS or CT. One week after the final immunization, the number of neutrophils (gated on: 7AAD^−^ CD45^+^ CD11c^+^ Ly6G^+^) and eosinophils (gated on: 7AAD^−^ CD45^+^ CD11c^+^ Siglec-F^+^) in the nasal passage were determined by flow cytometric analysis (*n* = 4 per group). Data are representative of two independent experiments and analyzed by one-way ANOVA (*p < 0.05; **p < 0.01; n.s., not significant).

Considering that the cytokines produced by T cells can cause neutrophilia and eosinophilia, which can lead to local inflammation, we examined the numbers of neutrophils and eosinophils in the nasal cavity of the nasally immunized mice. Consistent with the cytokine profiles, flow cytometry analysis revealed an increase in the numbers of neutrophils and eosinophils in the nasal cavity of CT group compared with the Mock or *Alcaligenes* LPS group (**Figure 6**). These results indicate that, unlike CT, *Alcaligenes* LPS did not induce local inflammation in the nasal cavity.

## Discussion

In this study, we showed the efficacy of *Alcaligenes* LPS as a nasal vaccine adjuvant to enhance antigen-specific respiratory and systemic immune responses including nasal and BALF IgA and serum IgG antibody responses (**Figures 1A** and **3**). Consistent with the elevation of IgA antibody responses (**Figures 1A** and **3**), our data indicated that nasal immunization with OVA plus *Alcaligenes* LPS induced GCs formation in the NALT and CLNs, where Tfh cells were also induced (**Figures 1B, C** and **2A, B**). In a previous study, we showed that *Alcaligenes* LPS stimulated bone marrow-derived DC (BMDC) or PP-derived DC to produce IL-6 (6), a cytokine involved in the differentiation of Tfh cells, Th17 cells, and IgA^+^ B cells (27–30). Collectively, these findings indicate that *Alcaligenes* LPS creates an immunological environment that promotes GC formation with Tfh cells and Th17 cells, which in turn induces antibody responses in the NALT and CLNs.

In the present study, although similar antibody responses were observed when mice were immunized with OVA plus *Alcaligenes* LPS or CT, the T cell responses induced by the adjuvants were different. Both adjuvants induced T cells producing IL-17, but the T cells induced by CT also expressed IFN-γ, whereas those induced by *Alcaligenes* LPS did not. IL-17 and IFN-γ–producing T cells are considered pathogenic because they induce severe inflammatory responses in autoimmune diseases (24, 31). Consistent with our present findings, studies by other groups have shown that CT induces IL-6, IL-1β, and IL-23 from DCs (32), which creates an environment that promotes the differentiation of pathogenic Th17 cells (33, 34). Regarding *Alcaligenes*, in our previous studies, we demonstrated that heat-killed *Alcaligenes* induces the production of IL-6, BAFF, TGF-β, and IL-10 when co-cultured with BMDCs, PP DCs, or murine PP cells (4, 6). Moreover, we also revealed that the stimulation of BMDCs with *Alcaligenes* lipid A resulted in the production of IL-6 and IL-23 (6, 23), the cytokines that are associated with differentiation of Th17 cell (34). However, in our previous studies, neither heat-killed *Alcaligenes* nor *Alcaligenes* lipid A induced BMDCs to produce IL-12 (6, 23), the cytokine that causes differentiation of Th1 cell (26). These characteristics plausibly led to the preferential differentiation of non-pathogenic Th17 cells by *Alcaligenes* LPS. Thus, it is likely that the production of IL-1β by antigen-presenting cells is the factor to determine which type of Th17 cells (pathogenic or non-pathogenic) is induced in our experimental condition. This is consistent with the results of a previous study, which showed that IL-1β is required for the pathogenicity of Th17 during intracellular bacterial infection (35).

In the present study, we also found that CT, but not *Alcaligenes* LPS, induced inflammation in the nasal cavity, which was characterized by increased infiltration of neutrophils and eosinophils. We also found that the levels of IL-5 and TNF-α production from T cells in the CT group were elevated but not in *Alcaligenes* LPS group. Consistent with this result, our previous studies showed that the production levels of TNF-α and nitric oxide (NO), an inflammatory molecule that induces TNF-α production, were lower in BMDCs treated with *Alcaligenes* LPS than in BMDCs treated with *E. coli* LPS (6, 36). IL-5 induces the differentiation of eosinophils (37) and interacts with IL-17 to promote the survival and degranulation of eosinophils, leading to tissue inflammation and damage (38). TNF-α upregulates vascular endothelial cell adhesion molecules, such as intercellular adhesion molecule 1 (ICAM-1), vascular adhesion molecule (VCAM-1), and E-selectin, thereby promoting the migration of neutrophils and eosinophils to sites of inflammation (37, 39). In addition, *Alcaligenes* LPS induced T cells secreting IL-10, which inhibits neutrophil recruitment by regulating the secretion of chemokines, such as CXCL9 and 12 and CCL3–5, 11, and 17 (40). Together, these findings indicate that *Alcaligenes* LPS did not induce inflammation because of lower numbers of T cells producing IL-5 or TNF-α and higher numbers of IL-10-producing T cells compared to CT, resulting in the migration of fewer eosinophils and neutrophils to the nasal cavity.

Regarding the immunological property of *Alcaligenes* LPS, our previous studies indicated that *Alcaligenes* LPS has little cytotoxic activity. Indeed, compared with *E. coli* LPS, *Alcaligenes* LPS showed lower endotoxin activity in the limulus amebocyte lysate test and caused only limited inflammatory reactions when intraperitoneally injected into mice, including lower levels of serum IL-6, less change in body temperature, and less damage to lung tissue with little infiltration of inflammatory cells, such as neutrophils and eosinophils (6). In addition, unlike *E. coli* LPS, *Alcaligenes* LPS showed little activity to induce apoptosis when co-cultured with BMDCs (36). In terms of IL-6 production from BMDCs, TLR4-deficient BMDCs did not respond to *Alcaligenes* LPS, whereas TLR2-deficient BMDCs produced comparable levels of IL-6 as wild type BMDCs (6). Further, *Alcaligenes* LPS did not act as a competitive inhibitor of *E. coli* LPS in the IL-6 production from BMDCs (6), collectively suggesting that *Alcaligenes* LPS acts as a weak agonist of TLR4, which is expressed in the nasal or lung tissues of mice (41, 42). This suggests that *Alcaligenes* LPS induced the immune responses also through combination of TLR4. As biochemical characteristics, the structure of LPS is mainly composed of lipid A, core oligosaccharide, and O antigen. Lipid A is considered to be the active center of LPS and acts as an agonist of TLR4/MD-2 complex. The activity as a TLR4 agonist is determined by several feature of lipid A structure. As for lipid A component in *Alcaligenes* LPS, a mixture of tetra- to hexa-acylated species was identified, and the lipid A with hexa-acylated species was composed of a bisphosphorylated glucosamine disaccharide backbone carrying 14:0 (3-OH) as primary and 12:0 (3-OH) and 10:0 as secondary fatty acids with distribution in a 3 + 3 fashion with respect to the disaccharide backbone, which were different with *E. coli* LPS whose lipid A has 4 + 2 symmetry and is composed of 14:0 (3-OH) as primary and 14:0 and 12:0 as secondary fatty acids (43). Although the other component of LPS, such as O-antigen, possibly plays some roles in the adjuvant activity of LPS (44), our previous studies implicated that the uniqueness of lipid A structure is the critical determinant of inflammatory activity.

In conclusion, *Alcaligenes* LPS showed efficacy as a nasal vaccine adjuvant to induce respiratory and systemic immune responses without inducing local inflammation *via* the induction of non-pathogenic Th17 responses and GC formation.

## Data Availability Statement

The original contributions presented in the study are included in the article/**Supplementary Material**. Further inquiries can be directed to the corresponding author. 

## Ethics Statement

The animal study was reviewed and approved by the guidelines of the Animal Care and Use Committee of the National Institutes of Biomedical Innovation, Health, and Nutrition (approval nos. DS27-47R13 and DS27-48R13).

## Authors Contributions

YW, KH, and JK contributed to conception and design of the study. YW, KH, KY, and AS planned and performed the experiments, analyzed the data. YW, KH, and JK wrote the first draft of the manuscript. AS, TN, YF, HK, and KF provided helpful discussion. All authors contributed to the article and approved the submitted version.

## Funding

This work was supported by the Ministry of Education, Culture, Sports, Science and Technology of Japan (MEXT)/Japan Society for the Promotion of Science KAKENHI (grant numbers 19KA3001 and 19K08955 to KH, 18H02674, 20H05697, 20K08534, 20K11560, 18H02150, 17H04134, 20H04117 to JK, 19K07617 and 20H03936 to TN, JP16K01914, JP20H04776, JP20K05749 to AS, JP15H05836, JP19KK0145, JP20H00404, JP20H05675 to KF), the Japan Agency for Medical Research and Development (AMED; grant numbers JP20ek0410062h0002, 20fk0108145h0001, JP20ak0101068h0004, and JP20gm1010006h004 to JK), The Ministry of Health and Welfare of Japan and Public/Private R&D Investment Strategic Expansion PrograM: PRISM (grant number 20AC5004 to JK), the Ministry of Health, Labour, and Welfare of Japan under (grant number JP19KA3001 to KH), Cross-ministerial Strategic Innovation Promotion Program: SIP (grant number 18087292 to JK), the Grant for Joint Research Project of the Institute of Medical Science, the University of Tokyo (to JK), the Ono Medical Research Foundation (to JK), the Canon Foundation (to JK), and Funding Program for Next Generation World Leading Researchers LR025 (YF).

## Conflict of Interest

The authors declare that the research was conducted in the absence of any commercial or financial relationships that could be construed as a potential conflict of interest.

## Supplementary Material

Supplementary Figure 1 Dose-dependent activity of LPS in the induction of nasal IgA responses. Mice were nasally immunized three times with OVA alone (Mock) or with OVA plus *Alcaligenes* LPS (1 or 10 μg) or *E. coli* LPS (1 or 10 μg). One week after the final immunization, Nasal wash was collected to determine levels of OVA-specific IgA by ELISA (*n* = 4 per group).

Supplementary Figure 2
*E. coli* LPS induced lymphopenia in the blood. Mice were nasally immunized with OVA alone (Mock) or with OVA plus Alcaligenes LPS (10 μg) or E. coli LPS (10 μg). 24 hours after immunization, blood samples were collected to measure the number of lymphocytes (n = 4 per group). Data are representative of two independent experiments and analyzed by one-way ANOVA (*p < 0.05; n.s., not significant).
