# Supplementary figures and images for: Lipopolysaccharide Derived From the Lymphoid-Resident Commensal Bacteria Alcaligenes faecalis Functions as an Effective Nasal Adjuvant to Augment IgA Antibody and Th17 Cell Responses
Source: Front Immunol. 2021 Jul 1;12:699349. doi: 10.3389/fimmu.2021.699349 (PMC8281128; doi:10.3389/fimmu.2021.699349)

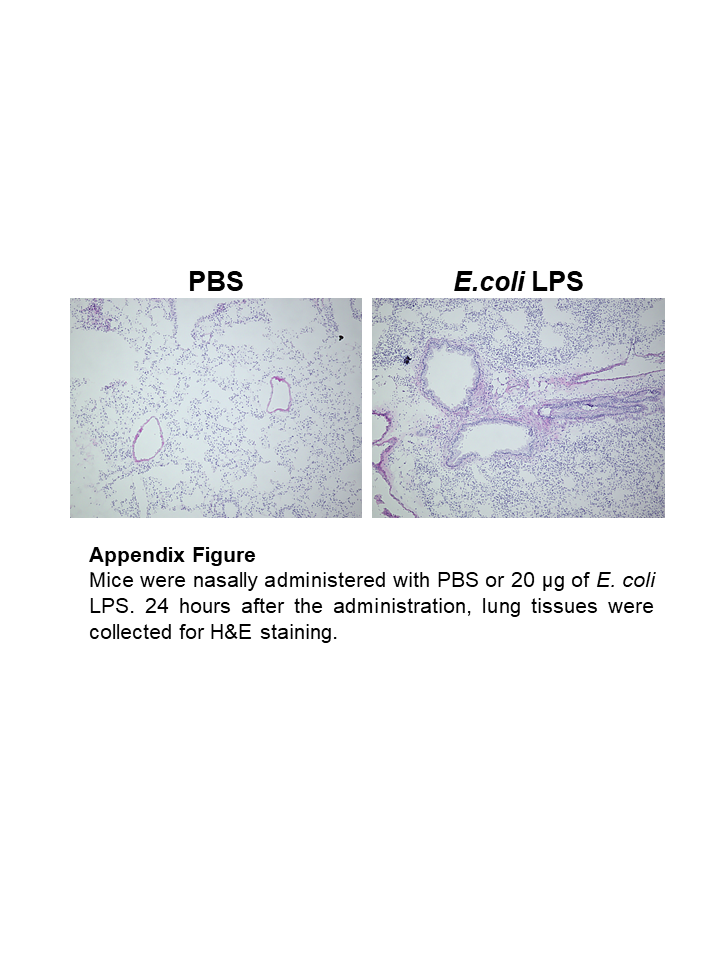

Supplement: Supplementary Figure 1 — Dose-dependent activity of LPS in the induction of nasal IgA responses. Mice were nasally immunized three times with OVA alone (Mock) or with OVA plus Alcaligenes LPS (1 or 10 μg) or E. coli LPS (1 or 10 μg). One week after the final immunization, Nasal wash was collected to determine levels of OVA-specific IgA by ELISA (n = 4 per group). [file Image_1.tif]

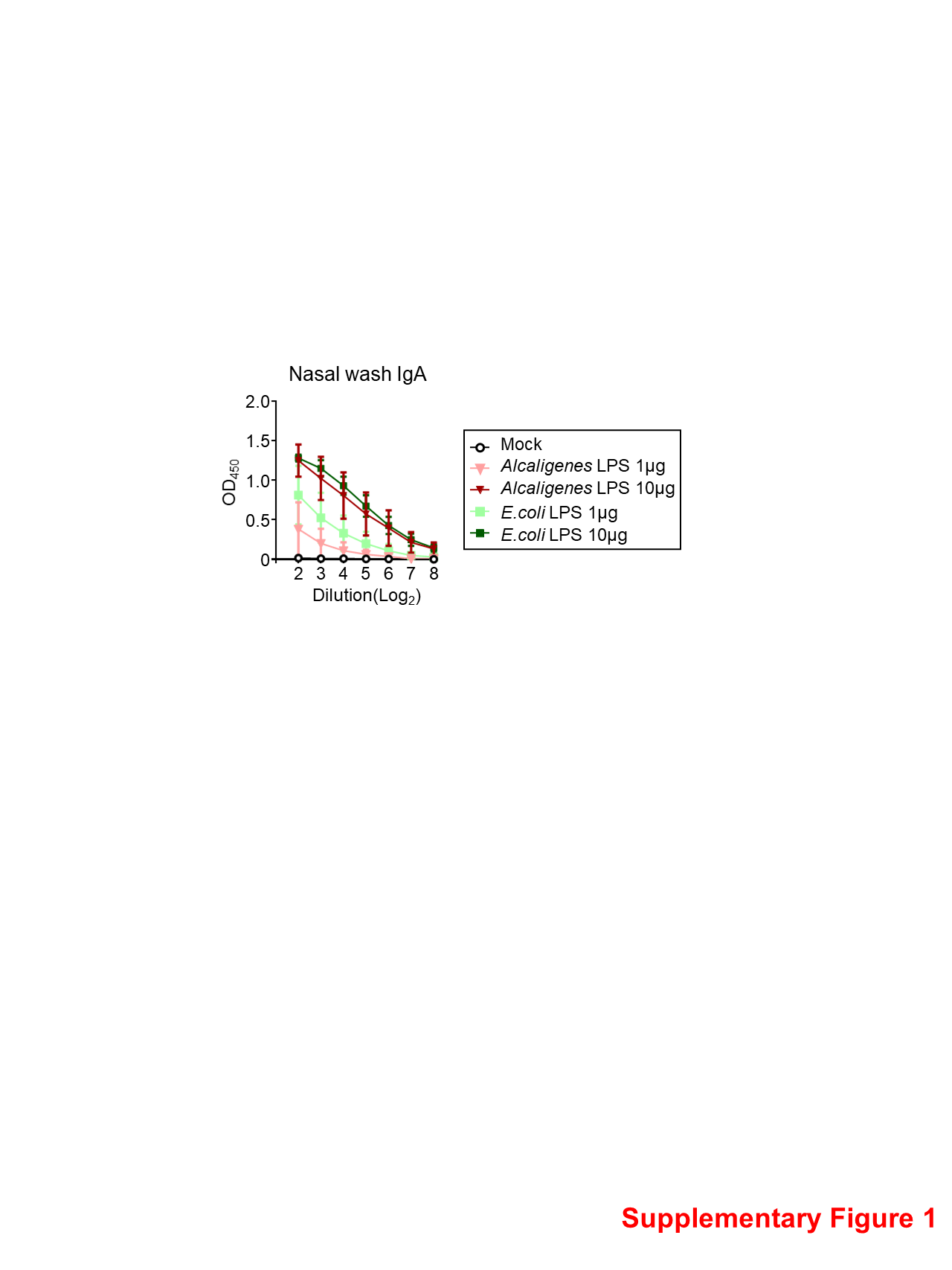

Supplement: Supplementary Figure 2 — E. coli LPS induced lymphopenia in the blood. Mice were nasally immunized with OVA alone (Mock) or with OVA plus Alcaligenes LPS (10 μg) or E. coli LPS (10 μg). 24 hours after immunization, blood samples were collected to measure the number of lymphocytes (n = 4 per group). Data are representative of two independent experiments and analyzed by one-way ANOVA (*p < 0.05; n.s., not significant). [file Image_2.tif]

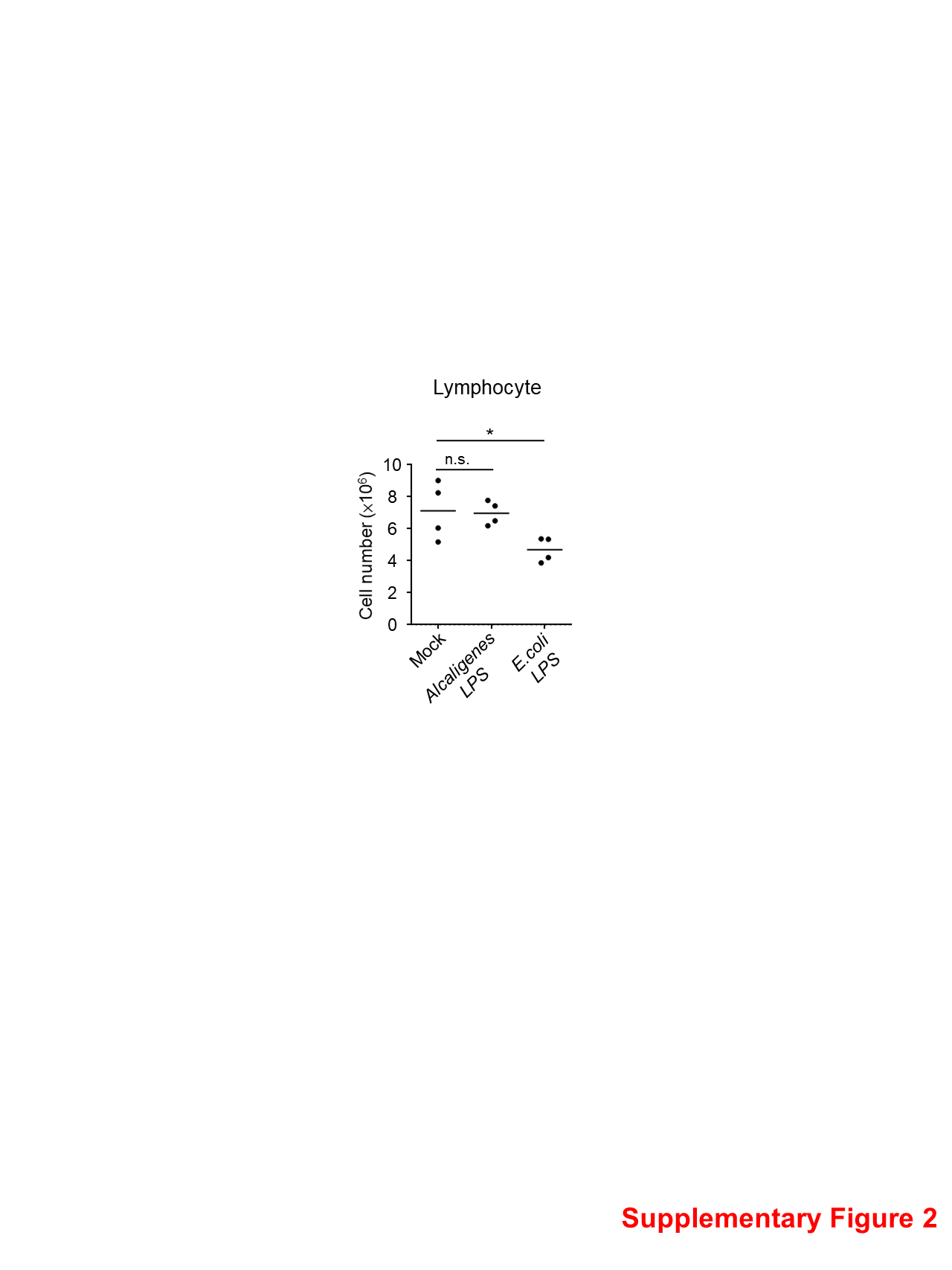

Supplement: Supplementary file 3 [file Image_3.tif]
